# Estimated mortality on HIV treatment among active patients and patients lost to follow-up in 4 provinces of Zambia: Findings from a multistage sampling-based survey

**DOI:** 10.1371/journal.pmed.1002489

**Published:** 2018-01-12

**Authors:** Charles B. Holmes, Izukanji Sikazwe, Kombatende Sikombe, Ingrid Eshun-Wilson, Nancy Czaicki, Laura K. Beres, Njekwa Mukamba, Sandra Simbeza, Carolyn Bolton Moore, Cardinal Hantuba, Peter Mwaba, Caroline Phiri, Nancy Padian, David V. Glidden, Elvin Geng

**Affiliations:** 1 Centre for Infectious Disease Research in Zambia, Lusaka, Zambia; 2 Johns Hopkins University, Baltimore, Maryland, United States of America; 3 Georgetown University, Washington, DC, United States of America; 4 Stellenbosch University, Cape Town, South Africa; 5 University of California, Berkeley, Berkeley, California, United States of America; 6 University of Alabama at Birmingham, Birmingham, Alabama, United States of America; 7 Lusaka Apex Medical University, Lusaka, Zambia; 8 Ministry of Health, Government of the Republic of Zambia, Lusaka, Zambia; 9 University of California, San Francisco, San Francisco, California, United States of America; Boston University, UNITED STATES

## Abstract

**Background:**

Survival represents the single most important indicator of successful HIV treatment. Routine monitoring fails to capture most deaths. As a result, both regional assessments of the impact of HIV services and identification of hotspots for improvement efforts are limited. We sought to assess true mortality on treatment, characterize the extent under-reporting of mortality in routine health information systems in Zambia, and identify drivers of mortality across sites and over time using a multistage, regionally representative sampling approach.

**Methods and findings:**

We enumerated all HIV infected adults on antiretroviral therapy (ART) who visited any one of 64 facilities across 4 provinces in Zambia during the 24-month period from 1 August 2013 to 31 July 2015. We identified a probability sample of patients who were lost to follow-up through selecting facilities probability proportional to size and then a simple random sample of lost patients. Outcomes among patients lost to follow-up were incorporated into survival analysis and multivariate regression through probability weights. Of 165,464 individuals (64% female, median age 39 years (IQR 33–46), median CD4 201 cells/mm^3^ (IQR 111–312), the 2-year cumulative incidence of mortality increased from 1.9% (95% CI 1.7%–2.0%) to a corrected rate of 7.0% (95% CI 5.7%–8.4%) (all ART users) and from 2.1% (95% CI 1.8%–2.4%) to 8.3% (95% CI 6.1%–10.7%) (new ART users). Revised provincial mortality rates ranged from 3–9 times higher than naïve rates for new ART users and were lowest in Lusaka Province (4.6 per 100 person-years) and highest in Western Province (8.7 per 100 person-years) after correction. Corrected mortality rates varied markedly by clinic, with an IQR of 3.5 to 7.5 deaths per 100 person-years and a high of 13.4 deaths per 100 person-years among new ART users, even after adjustment for clinical (e.g., pretherapy CD4) and contextual (e.g., province and clinic size) factors. Mortality rates (all ART users) were highest year 1 after treatment at 4.6/100 person-years (95% CI 3.9–5.5), 2.9/100 person-years (95% CI 2.1–3.9) in year 2, and approximately 1.6% per year through 8 years on treatment. In multivariate analysis, patient-level factors including male sex and pretherapy CD4 levels and WHO stage were associated with higher mortality among new ART users, while male sex and HIV disclosure were associated with mortality among all ART users. In both cases, being late (>14 days late for appointment) or lost (>90 days late for an appointment) was associated with deaths. We were unable to ascertain the vital status of about one-quarter of those lost and selected for tracing and did not adjudicate causes of death.

**Conclusions:**

HIV treatment in Zambia is not optimally effective. The high and sustained mortality rates and marked under-reporting of mortality at the provincial-level and unexplained heterogeneity between regions and sites suggest opportunities for the use of corrected mortality rates for quality improvement. A regionally representative sampling-based approach can bring gaps and opportunities for programs into clear epidemiological focus for local and global decision makers.

## Abstract 2

To improve estimates of mortality for patients who initiate HIV treatment, Charles Holmes and colleagues use a multistage sampling approach to find and trace health outcomes for patients lost to follow-up after starting antiretroviral therapy in Zambia.

## Author summary

**Why was this study done?:**

- Previous studies from cohorts in South Africa and parts of East Africa have suggested that site-level reporting of mortality is incomplete.
- We wanted to understand the degree to which this phenomenon was impacting HIV outcomes at a broader scale, in this case at the provincial level in Zambia, a country with one of the highest burdens of HIV.
- We also wanted to gain an in-depth understanding of differences between the outcomes of clinical care sites in order to assess the role of mortality as a potential quality improvement target.

**What did the researchers do and find?:**

- From a source population of patients in 4 provinces (Lusaka, Southern, Eastern, and Western) who visited government-operated HIV treatment sites in these provinces, we conducted a multistage sampling approach of a stratified selection of sites and a random sample of patients lost to follow-up.
- Lost patients were traced and their vital status was ascertained, which was used to enable a corrected regionally representative estimate of survival after starting antiretroviral therapy (ART) as well as corrected site-specific mortality estimates.
- Of 165,464 individuals, the 2-year cumulative incidence of mortality increased from 1.9% to 7.0% for all ART users and from 2.1% to 8.3% for new ART users, and provincial-level mortality rates rose 3- to 8-fold once corrected for true outcomes.
- Being late (>14 days late for appointment) or lost (>90 days late for an appointment) was associated with death.

**What do these findings mean?:**

- Deaths are under-reported within the Zambian HIV program, and mortality rates are highly variable across sites and provinces.
- Our findings enable national- and global-level policy makers to correct existing underestimates of mortality, link these data to quality improvement efforts, and reprioritize interventions to target regional and site-level reductions in mortality as a goal of HIV programs.
- We have also established that this methodology is feasible for use as a representative surveillance tool for accurate monitoring of provincial and potentially national levels of mortality, even as vital status registries and data systems are further developed and strengthened.

## Introduction

As the global response to HIV moves towards universal treatment, with the intent of ending AIDS as a public health crisis by 2030, a rigorous understanding of contemporary program effectiveness demonstrates progress as well as identifies important opportunities for improvement [1]. Although other metrics also contain important information, mortality captures program effectiveness better than any other single process or clinical outcome. For example, although plasma HIV RNA suppression is a critical measure of HIV control and uncontrolled viremia indicates immunological deterioration, suppressed HIV RNA does not reflect benefits from other aspects of care such as isoniazid preventative therapy, management of side effects, or treatment of opportunistic infections. Of note, even 90-90-90 [2]—a centrally important set of metrics of national program performance—does not include monitoring of a program’s success in saving lives.

Despite the importance of mortality, relatively little data exist about long-term survival after initiation of HIV treatment in lower- and middle-income countries [3]. In South Africa, a new National Population Register for recording vital status has enabled an advanced understanding of mortality, but most high-burden settings lack such registries [4,5]. Routine monitoring and evaluation of treatment programs also fail to capture most deaths because they often happen outside of the health system in patients considered “lost to follow up” [6,7]. A nomogram has been proposed that incorporates the results of some of these tracing studies and uses the fraction of individuals lost to assess mortality, but it is not always highly accurate at site-level estimates [8]. Efforts have been undertaken to understand the “true” mortality rates through tracing random samples of patients lost to follow-up in convenience samples of clinics in multiple studies [9–11], but this approach has only been examined in the context of a limited number of conveniently sampled facilities. Mathematical models for the Joint United Nations Programme on HIV/AIDS (UNAIDS) and others report on mortality but depend on a number of assumptions and limited underlying epidemiological data [12].

In order to rigorously assess mortality, we apply for the first time, to our knowledge, a multistage sampling design to obtain representative estimates of mortality in 4 provinces in Zambia as well as site-level estimates with enough precision to identify site-to-site variation. This approach offers a rigorous estimate of (1) the magnitude of deaths after starting antiretroviral therapy (ART) among those already on treatment at the start of the study and among new treatment initiates, (2) when deaths occur, (3) which groups are at highest risk of death, and (4) whether these factors differ by region, facility, or other important factors such as prior healthcare utilization patterns. Both the approach and findings have implications for assessment of program effectiveness and program improvement efforts.

## Methods

The protocol and study were approved by the University of Zambia Biomedical Research Ethics Committee (UNZABREC) (004-06-14) and the IRB of the University of Alabama, Birmingham, School of Medicine (F160122006), and the submitted protocol is available in the supplemental material. In this paper, we used a number of analytic innovations not specified in the analysis protocol, including the use of a modified Lorenz curve to depict the distribution of deaths across facilities and survival analyses of mortality in which deaths are classified by retention histories. In addition, analyses of pretreatment experience as specified in the protocol are also planned but not included in this paper.

### Population and sampling

The target population for this analysis is the contemporary population of HIV-infected adults on ART in Zambia. We considered “contemporary” to be patients who had a clinical encounter while on ART (including the ART initiation visit) in the 24 months before the evaluation, and they are referred to as “all ART users” and provide the most complete picture of treatment outcomes. We also designate a subpopulation of this group as “new ART users,” i.e., those who initiated ART during the 24-month period prior to the evaluation—this group is more readily comparable to outcomes from other analyses of those starting ART. Our source population consisted of patients in 4 provinces (Lusaka, Southern, Eastern, and Western) who visited government-operated HIV treatment sites in these provinces, each of which receives technical assistance support from Centre for Infectious Disease Research in Zambia (CIDRZ), a local Zambian organization.

The analysis population was enumerated using a multistage sampling approach to yield estimates representative of the entire population of patients on treatment as well as to enable comparison, through stratification, across 4 provinces and 3 facility types (hospital, urban health center, and rural health center). We selected using probabilities proportional to size a minimum of 2 to 10 facilities from each of 12 strata defined by facility type and province for a total of 32 facilities. In each selected facility, we enumerated all adults on ART who made a visit in the previous 24 months and identified loss to follow-up from this cohort (defined as >90 days late at the time of sampling). From patients lost to follow-up, we selected a simple random sample for tracing inverse proportional to size. This approach seeks to enable both a regionally representative estimate of survival after starting ART and site-specific estimates with approximately 95% confidence estimates from 5% to 15% in a setting where mortality is estimated to be 10% [9]. We additionally used simulation to optimize the sampling strategy to achieve these aims. A diagram detailing the sampling process and tracing outcomes is presented in the supplementary materials (S1 Fig).

### Procedures and measurements

As described in previous work, we used data from an electronic medical records system to enumerate the cohort and to obtain sociodemographic and clinical data. Peer health workers traced lost patients intensively in the community to ascertain vital status [13]. Tracing included in-depth review of paper and electronic medical records, phone calls, and in-person tracing in the community. Tracers used bicycles, public transportation, study vehicles, or motorcycles as needed. A minimum of 3 tracing attempts was required. If patients were found to be dead, death dates, cause of death, and location of death were solicited from family and other close contacts. The list of patients to be traced was ranked in random order and released in blocks of 25 to each facility to preserve the benefits of a random sample.

### Analysis

We estimated the cumulative incidence of mortality using the Kaplan Meier (KM) approach as well as simple mortality rates. In KM estimates, we treated time zero as the date of ART initiation. Patients who started ART before the observation period were left-truncated, which yields an estimate of survival after ART initiation during the 2-year period of observation of this study that is contingent on surviving into this period. This approach is analogous to life expectancy estimates. Mortality estimates were carried out for all ART users as well as stratified by new ART initiators during the 2-year observation period and those already on ART at the start of the current period. These analyses are intended to convey the totality of the contemporary experience of the patient population, including both that of those on treatment for extended periods of time and that of important subpopulations. We carried out mortality estimates both using only deaths known to the program before tracing and after incorporating findings from tracing lost patients to “correct” estimates through use of probability weights. These individual weights were combined with facility-level weights for estimates in the entire patient population.

In additional survival analyses, we used the Aalen Johansen method to estimate the cumulative incidence of death events classified by their current and previous engagement history using the reweighted data. We defined patients as having “died in care” if they had died within 30 days of their last visit; the remaining patients were classified as having “died out of care.” In addition, patients were categorized as “previously lost” if they had had at least 1 episode of being out of care for more than 90 days after an appointment in the past and returned to care prior to our study sampling and “previously late” if they had at least 1 episode of being late for an appointment by more than 30 days but less than 90 days prior to our study sampling. These categories yielded 6 engagement states for the analysis [14,15]. We examined the sociodemographic (e.g., sex and age), clinical (e.g., CD4 level at ART initiation), and health systems factors (e.g., facility size) associated with mortality through simple stratification and multivariate Poisson regression. Analytic weights inverse to the probability of missingness were used to address missing predictor side variables under the missing-at-random assumption [16]. We quantified site-to-site variability in mortality by examining the distribution of site-specific mortality estimates. In order to summarize the unequal contribution to total mortality across facilities, we used a modification of the Lorenz curve in which the *y*-axis displays the cumulative proportion of excess deaths and the *x*-axis the cumulative population proportion after adjustment for patient (e.g., sex and CD4 level at ART initiation) as well as facility factors (e.g., facility volume) [17]. Excess deaths are defined as deaths above a threshold of 1% at 2 years by facility—the approximate death rate in cohorts from Europe of stable patients used to represent “ideal” outcomes [18]. The Lorenz analysis was restricted to new ART initiators in order to minimize the effect of local, clinic-specific distribution of patient time of treatment.

We used 3 approaches to characterize deaths over time. First, we graphically displayed the retention history with all patients who died represented by a single horizontal bar. The length of the bar represents time since ART initiation, and colors represent time spent in care, late for care (>30 days and <90 days late for a visit), or lost (>90 days late for last appointment). Second, we examined mortality rates in each year after ART initiation among all patients as well as only among those patients who had made a visit in the last 2 years (and therefore survived into the current era) and tested for significance in the change over time. Third, we added time-varying covariates—(1) engaged, never late or lost; (2) engaged, but with a history of being late or lost; (3) late without a history of previous episodes of being late or lost; (4) late with a history of being lost; and (5) being lost (defined as being >90 days since last appointment)—in multivariable regression to capture the association between lapses in retention states and mortality. Based on the revised estimates, we quantified the fraction of deaths that fell into each category of visit histories and in each of the segments of time after ART initiation. We calculated the “population attributable fraction” of all deaths that occurred in each of these states—an estimate of how much mortality could be avoided if all measured characteristics were set at the lowest level of risk.

## Results

### Patient characteristics

**Fig 1:**
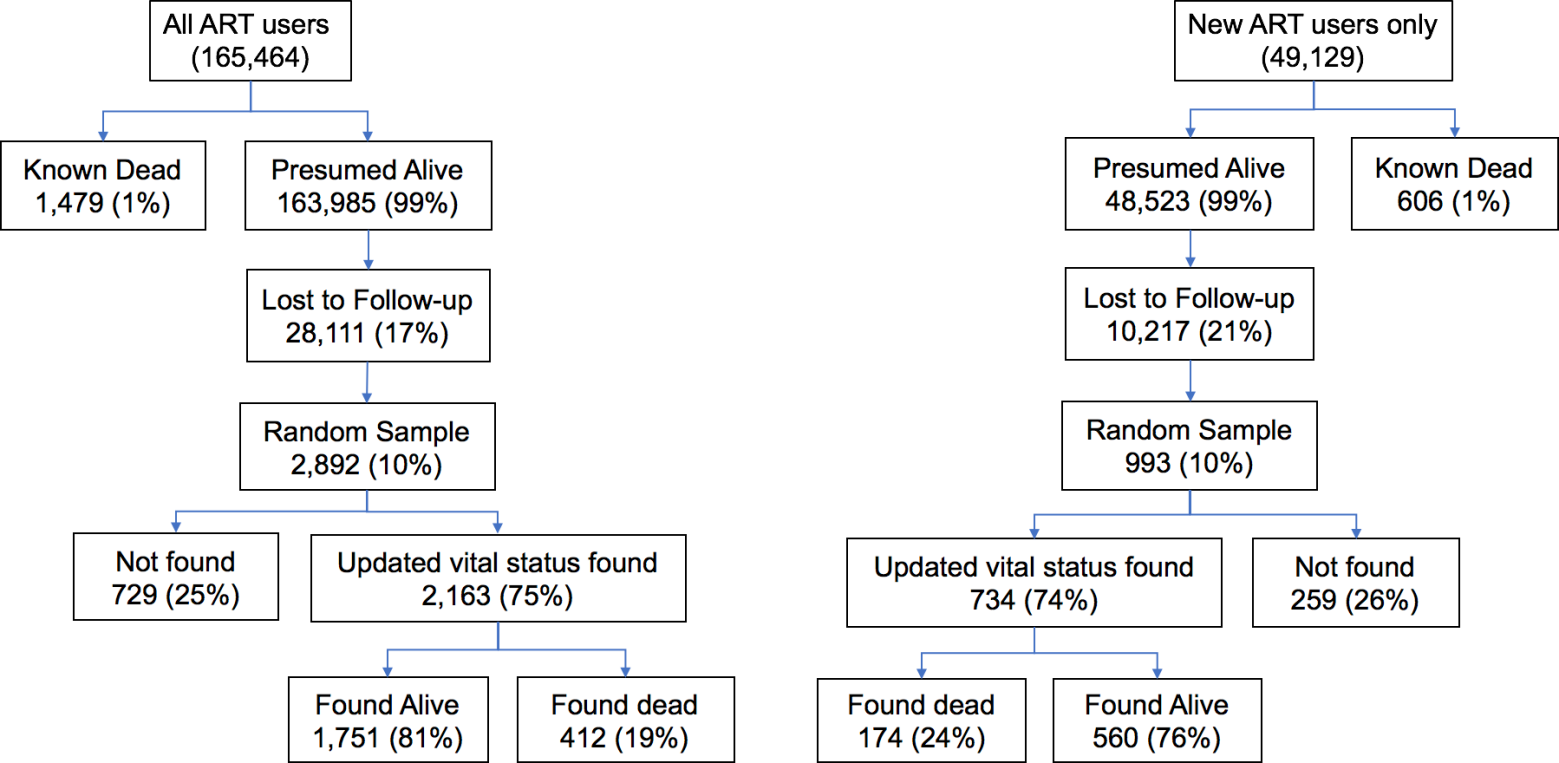
Flowchart depicting tracing outcomes among all patients on antiretroviral therapy (ART) and the subset of new ART initiators during the 2-year study period at 64 facilities.

In a network of 64 HIV delivery facilities operated by the Zambian Ministry of Health and supported by CIDRZ across 4 provinces, we identified a total of 165,464 individuals on ART and who had made a visit in the 2 years before the project period (i.e., 1 August 2013 to 31 July 2015), which included 49,129 individuals newly initiating ART during that period and 116,335 individuals already on treatment at the opening of the observation period (Fig 1).

In the entire cohort (Table 1), 64% were female, the median age was 39 years (IQR 33–46), 56% attended urban facilities, about half were from Lusaka Provence (52%), median CD4 at ART initiation was 201 cells/mm^3^ (IQR 111–312), and 58% were WHO stage 1 or 2 at ART initiation. New ART users were similar demographically but had a higher median CD4 level at ART initiation of 262 cells/mm^3^ (IQR 138–288), and 65% were WHO stage 1 or 2 at enrollment. The median time on ART was 1,142 days (IQR 390–2,139) for all ART users in the cohort and 255 days (IQR 63–407) in new ART starters. The median date of ART initiation for all users was 14 February 2012 (IQR 03 June 2009–23 January 2014) and for new ART users was 24 July 2014 (IQR 3 February 2014–23 December 2014).

**Table 1:** Descriptive characteristics of the study cohort.

		All ART users				New ART users			
		All	Lost	Traced	Vital status ascertained	All	Lost	Traced	Vital status ascertained
		*N* (%)	*N* (%)	*N* (%)	*N* (%)	*N* (%)	*N* (%)	*N* (%)	*N* (%)
**Total patients**		165,464	28,111	2,892	2,163	49,129	10,217	993	734
**Gender**	Female	105,745 (64)	16,870 (60)	1,705 (59)	1,254 (58)	30,858 (63)	6,236 (61)	596 (80)	428 (58)
	Male	59,719 (36)	11,241 (40)	1,187 (41)	909 (42)	18,271 (37)	3,981 (39)	397 (40)	306 (42)
**WHO stage at enrolment**	Stage 1	62,116 (38)	10,690 (38)	1,059 (37)	783 (36)	23,544 (48)	4,552 (45)	433 (44)	321 (44)
	Stage 2	33,288 (20)	5,080 (18)	588 (20)	445 (21)	8,574 (17)	1,605 (16)	174 (18)	130 (18)
	Stage 3	48,738 (29)	8,453 (30)	766 (26)	589 (27)	8,779 (18)	2,294 (22)	180 (18)	138 (19)
	Stage 4	5,497 (3)	1,006 (4)	130 (4)	94 (4)	777 (2)	241 (2)	32 (3)	22 (3)
	Unknown	15,825 (10)	2,882 (10)	349 (12)	252 (12)	7,455 (15)	1,525 (15)	174 (18)	123 (17)
**Year of enrolment**	2004–2006	16,198 (10)	1,723 (6)	142 (5)	93 (4)	230 (0)	58 (1)	4 (0)	2 (0)
	2007–2009	41,050 (25)	5,538 (20)	570 (20)	435 (20)	1,505 (3)	328 (3)	32 (3)	20 (3)
	2010–2012	53,594 (32)	9,148 (33)	1,015 (35)	758 (35)	5,341 (11)	1,053 (10)	109 (11)	73 (10)
	2013–2015	54,622 (33)	11,702 (42)	1,165 (40)	877 (41)	42,053 (86)	8,778 (86)	848 (85)	639 (87)
**Province**	Eastern	29,701 (18)	3,523 (13)	553 (19)	464 (21)	9,234 (19)	1,163 (11)	170 (17)	136 (19)
	Lusaka	86,688 (52)	17,754 (63)	1,284 (44)	884 (41)	25,644 (52)	6,672 (65)	444 (45)	305 (42)
	Southern	24,864(15)	2,714 (10)	507 (18)	384 (18)	6,564 (13)	944 (9)	196 (20)	151 (21)
	Western	24,211 (15)	4,120 (15)	548 (19)	431 (20)	7,687 (16)	1,438 (14)	183 (18)	142 (19)
**Facility type**	Rural	16,547 (10)	3,163 (11)	633 (22)	536 (25)	5,442 (11)	1,198 (12)	227 (23)	189 (26)
	Urban	92,216 (56)	17,667 (63)	1,476 (51)	1,047 (48)	28,053 (57)	6,674 (65)	540 (54)	386 (53)
	Hospital	56,701 (34)	7,281 (26)	783 (27)	580 (27)	15,634 (32)	2,345 (23)	226 (23)	159 (22)
**Disclosure of HIV status at treatment initiation**	No	2,580 (2)	642 (2)	63 (2)	41 (2)	1,293 (3)	355 (3)	26 (3)	18 (2)
	Yes	142,021 (86)	24,027 (85)	2,472 (85)	1,844 (85)	43,329 (88)	8,846 (87)	871 (88)	639 (87)
	Unknown	20,863 (13)	3,442 (12)	357 (12)	278 (13)	4,507 (9)	1,016 (10)	96 (10)	77 (10)
**Highest education level**	None	9,660 (6)	1,674 (6)	226 (8)	170 (8)	3,100 (6)	626 (6)	90 (9)	63 (9)
	Lower-mid basic	48,175 (29)	7,606 (27)	813 (28)	599 (28)	14,462 (29)	2,785 (27)	263 (26)	195 (27)
	Upper basic/secondary	62,154 (38)	11,542 (41)	1,098 (38)	833 (39)	19,894 (40)	4,365 (43)	392 (39)	295 (40)
	College/university	6,398 (4)	1,107 (4)	113 (4)	98 (5)	1,831 (4)	374 (4)	42 (4)	35 (5)
	Unknown	39,077 (24)	6,182 (22)	642 (22)	463 (21)	9,842 (20)	2,067 (20)	206 (21)	146 (20)
**Marital status**	Single	14,965 (11)	3,130 (14)	320 (13)	239 (13)	5,059 (13)	1,224 (15)	135 (17)	108 (18)
	Married	86,091 (63)	14,422 (63)	1,493 (63)	1,147 (63)	25,988 (66)	5,281 (64)	514 (64)	386 (64)
	Divorced	16,958 (12)	3,103 (14)	342 (14)	250 (14)	5,227 (13)	1,131 (14)	93 (12)	65 (11)
	Widowed	16,125 (12)	2,211 (10)	228 (10)	174 (10)	3,397 (9)	603 (7)	64 (8)	46 (8)
	Unknown	2,578 (2)	0	0	0	0	0	0	0
** **									
**Age;** years (median [IQR])		39 (33–46)	36 (30–43)	37 (31–44)	37 (31–44)	35 (29–42)	33 (28–40)	34 (28–41)	34 (29–41)
**Enrolment cd4 count;** cells/mmol (median [IQR])		224 (119–357)	220 (115–354)	220 (111–362)	217 (112–352)	281 (145–435)	254 (125–409)	268 (121–423)	256 (119–412)
**Initiation CD4 count;** cells/mmol (median [IQR])		201 (111–312)	201 (108–318)	200 (103–313)	199 (105–309)	262 (138–388)	238 (116–368)	226 (102–364)	225 (111–370)
**Time on ART;** days (median [IQR])		1,142 (390–2,139)	535 (98–1492)	102 (89–111)	79 (64–91)	225 (63–407)	49 (1–180)	46 (1–170)	59 (0–185)
**Facility size;** number of patients per facility (median [IQR])		7,566 (4,060–60,498)	721 (411–1,434)	592 (104–1,496)	611 (119–1,508)	1,082 (722–1,959)	258 (156–512)	35 (30–364)	26 (22–31)
**ART start date;** date (median [IQR])		14 Feb 2012 (03 Jun 2009–23 Jan 2014)	23 Jan 2013 (21 Jun 2010–12 Feb 2014)	16 Dec 2012 (29 Jun 2010–17 Feb 2014)	28 Nov 2012 (30 Jun 2010–24 Feb 2014)	24 Jul 2014 (3 Feb 2014–23 Dec 2014)	02 May 2014 (06 Dec 2013–09 Sep 2014)	14 May 2014 (27 Dec 2013–24 Sep 2014)	21 May 2014 (02 Jan 2014–25 Sep 2014)

ART, antiretroviral therapy.

### Naïve and corrected mortality estimates

**Fig 2:**
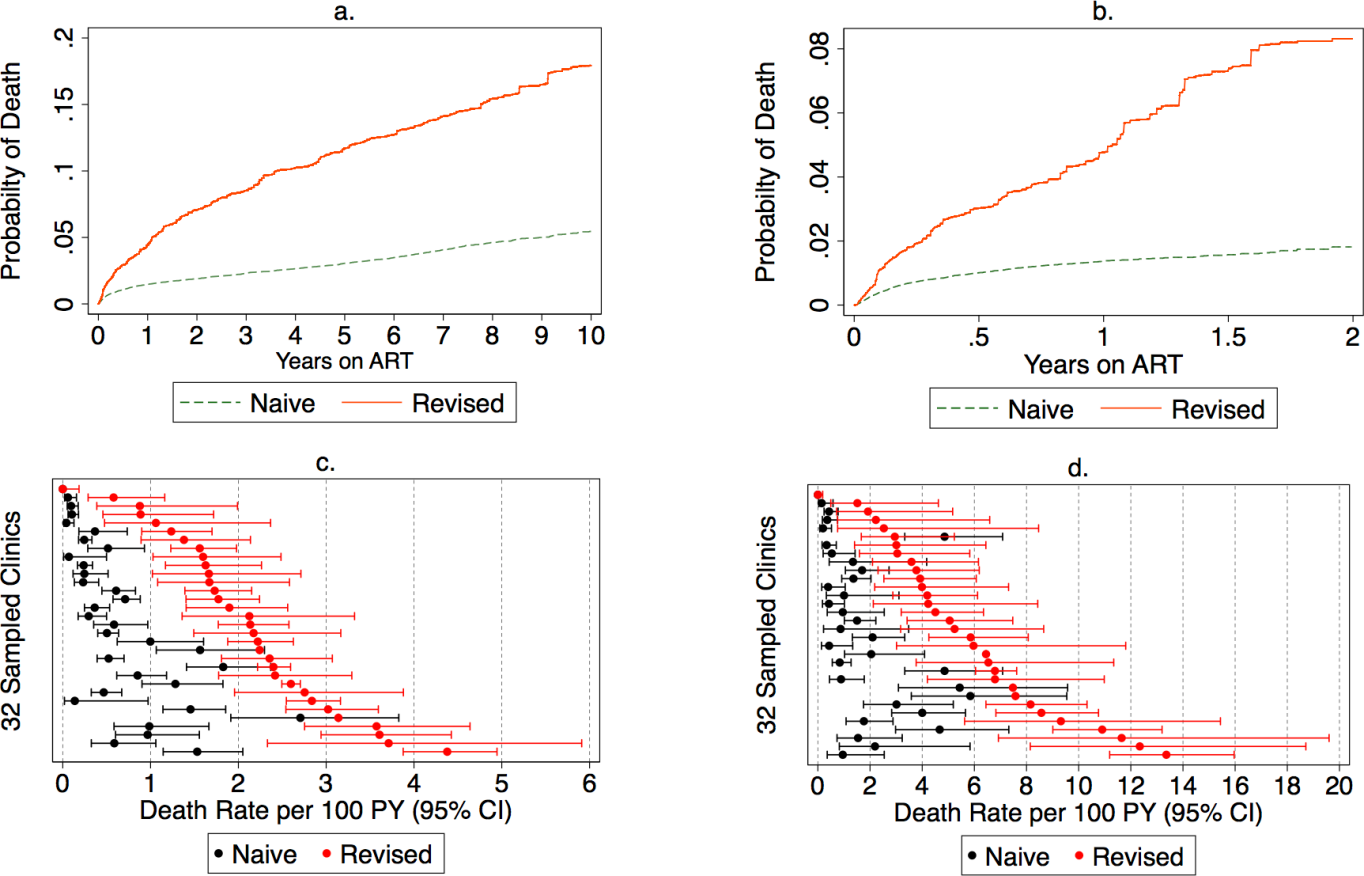
**Naïve and revised mortality estimates among all antiretroviral therapy (ART) users (*N* = 165,464) and new ART users (*N* = 49, 129) in (A) cumulative incidence of mortality among all ART users.** (B) Cumulative incidence of mortality among new ART users. (C) Mortality rate among all ART users. (D) Mortality rate among new ART users. PY, person-year.

Routine program data before the tracing exercise suggested that 1% of all patients had died. A total of 17% of all patients were lost to follow-up, and this fraction lost was highly variable across clinic sites (IQR 10%–31%). Among the 28,111 lost patients from the entire cohort, we made an attempt to trace 2,892 (10%). Tracing efforts led to updated vital status in 2,163/2,892 (75%) of patients, but ascertainment of vital status varied from 54% to 93% across all sites (S1 Table). Among patients lost to follow-up from the entire cohort, 17% (95% CI 15%–19%) had died. Among patients lost from the group newly starting ART during the 2-year observation period, 21% had died (95% CI 17%–25%). After incorporating outcomes from tracing into underlying data known to the program, we found the 2-year cumulative incidence of mortality among all ART users increased from a naive estimate of 1.9% (95% CI 1.7%–2.0%) to a sample-revised estimate of 7.0% (95% CI 5.7%–8.4%) (Fig 2). Among new ART users, sampling changed the cumulative incidence of mortality at 2 years from a naïve estimate of 2.1% (95% CI 1.8%–2.4%) to a revised estimate of 8.3% (95% CI 6.1%–10.7%) (Fig 2). Facility-level revised mortality estimates ranged widely, with an IQR from 1.6 to 2.6 deaths per 100 person-years among all patients and an IQR of 3.5 to 7.5 deaths per 100 person-years and a high of 13.4 deaths per 100 person-years among new ART initiators (Fig 2). Most facilities showed marked differences between naïve and revised estimates, with 1 site showing a 23-fold difference in mortality among all patients and another site showing a 14-fold difference for new ART users.

**Fig 3:**
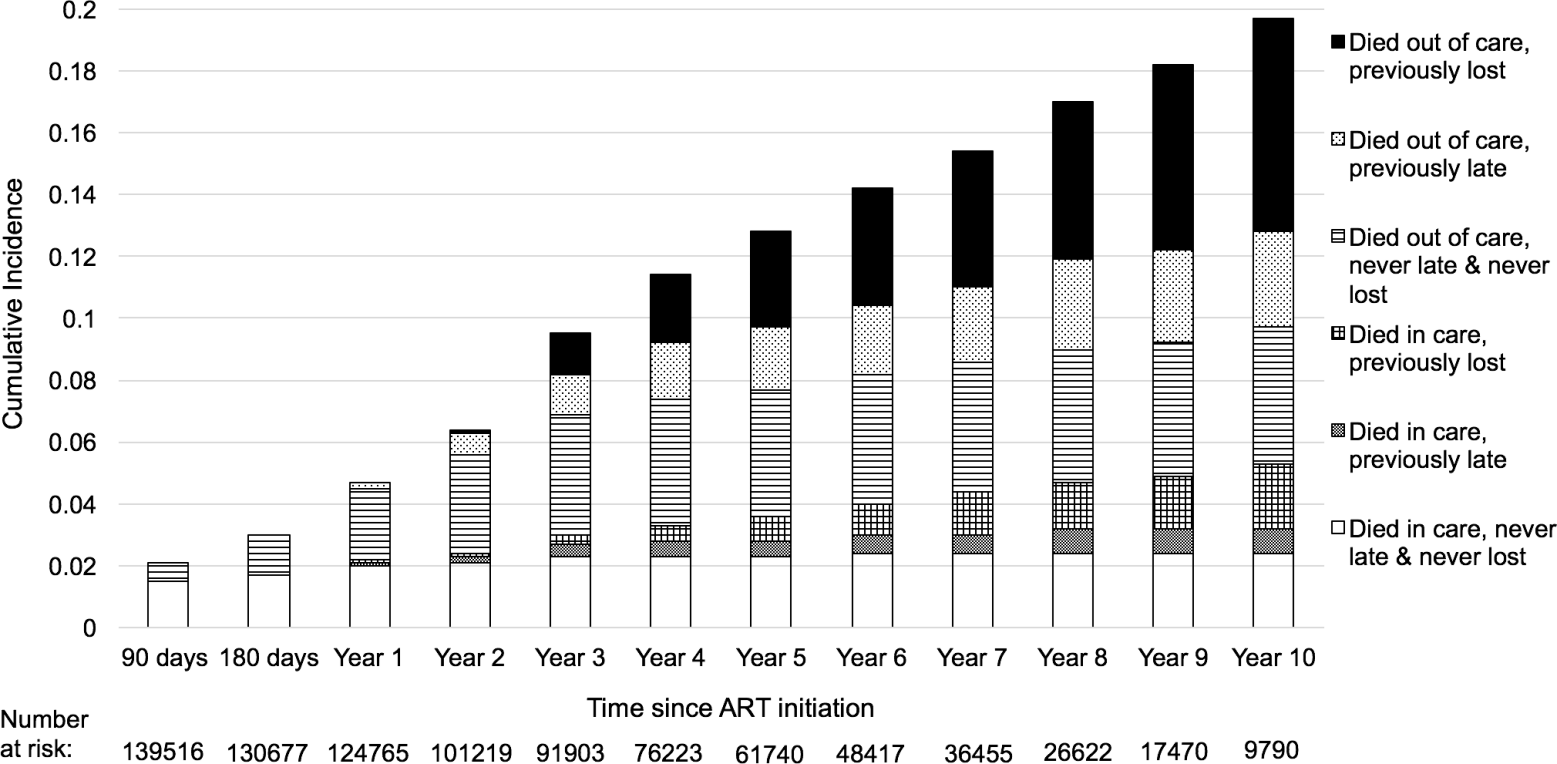
Proportion of patients who died after starting antiretroviral therapy (ART) by engagement status, with left truncation for individuals on ART before the study observation period (*N* = 165,464). Lost was defined as having been more than 90 days late for a visit, and late was defined as being 15–90 days late for a visit.

The deaths during the first year on treatment occur mostly in persons who have not missed appointments. There is an increase over time in the fraction of deaths that occur in patients who are either late for a visit or who have a history of being late or lost from care (Fig 3). The cumulative incidence estimates and confidence intervals for this analysis are presented in the supplementary materials (S2 Table). Furthermore, general categories of reported causes of death were available for about half of the patients who died (S3 Table).

### Regional and facility-level mortality rates

**Fig 4:**
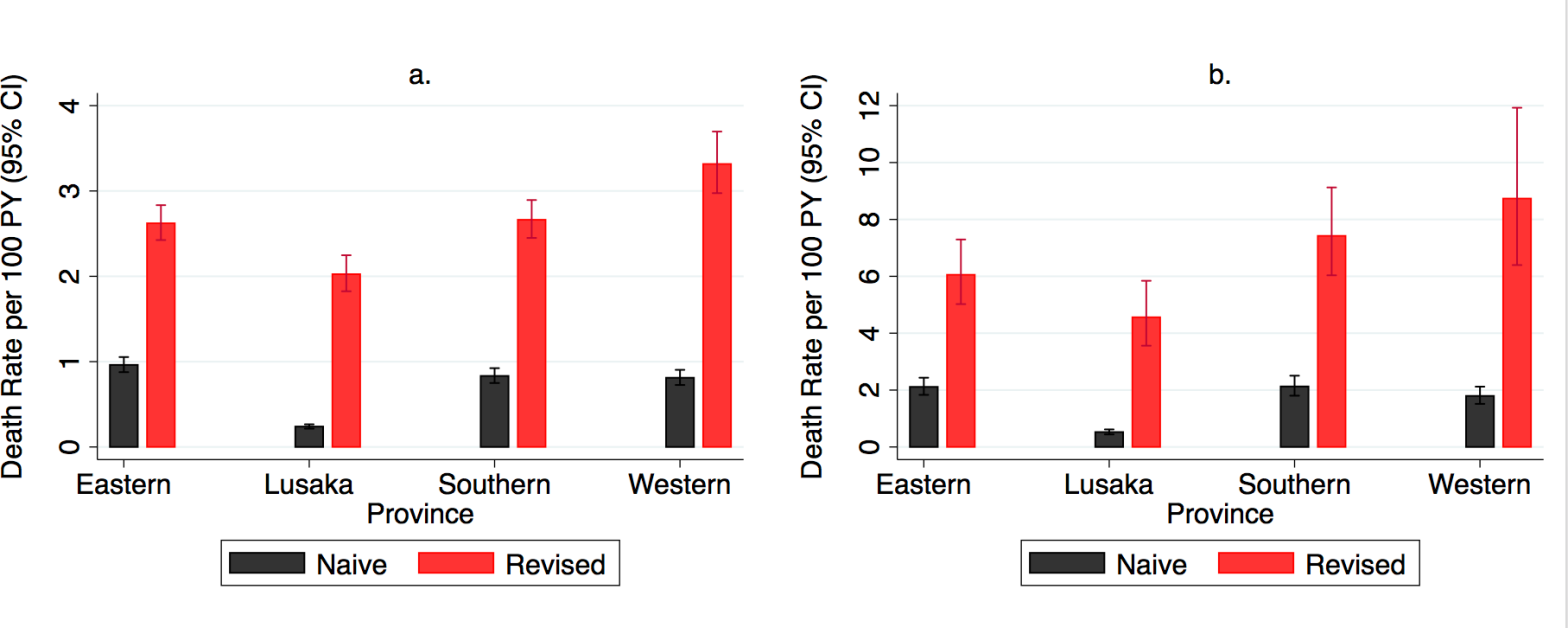
**Naïve and revised mortality rates by province in (A) all antiretroviral therapy (ART) users (*N* = 165,464) and (B) new ART users (*N* = 49,129).** PY, person-year.

**Fig 5:**
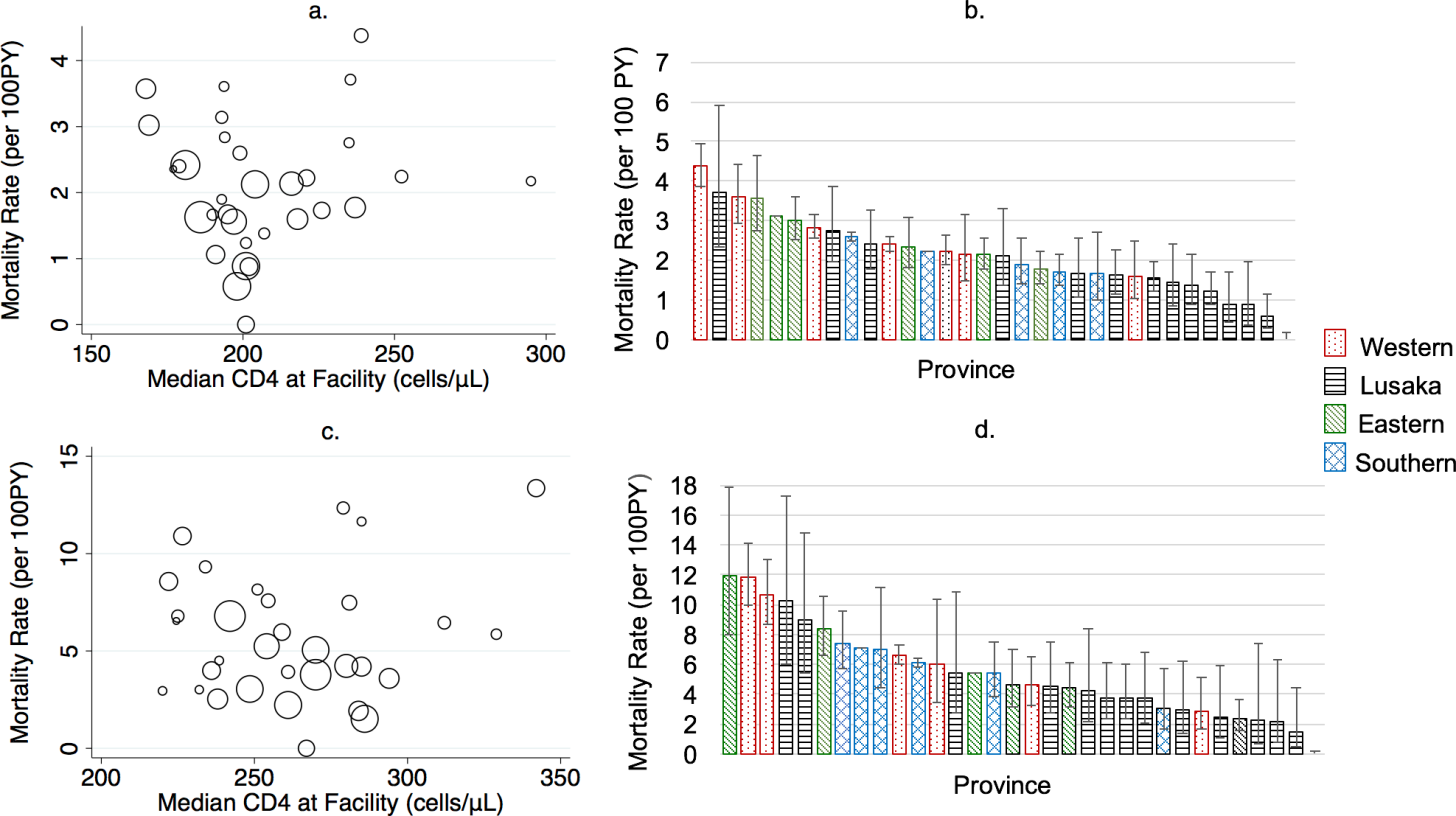
**(A) Facility-level mortality rate by median CD4 at treatment initiation and (B) facility-level mortality estimates by province among all antiretroviral therapy (ART) users (*N* = 165,464); (C) facility-level mortality rate by median CD4 and (B) facility-level mortality rate by province in new ART users (*N* = 49,129).** PY, person-year.

Mortality varied markedly across geographical areas and facilities. At the provincial level, revised estimates of mortality were higher than naïve estimates, ranging in all ART users from 3-fold higher in Eastern and Southern Province to 8-times higher in Lusaka Province and in new ART users from 3-fold higher in Eastern Province to 9-fold higher in Lusaka Province (Fig 4, S4 Table). The highest absolute mortality rates, prior to revision, were in Eastern Province (all ART users) and Southern Province (new ART users), whereas the highest revised mortality rates were in Western Province, followed by Southern, Eastern, and Lusaka Provinces, for both all ART users and new ART users. Facility-level mortality estimates were highly variable even within province and across facilities with similar median CD4 levels at ART initiation (Fig 5).

Multivariable regression models using patient and facility characteristics at ART initiation found that male sex, CD4 level (≥200 cells/μL) at ART initiation, and higher WHO stage were associated with higher mortality in new ART users, whereas facility size and type (health center) were protective. For those already on ART at the start of observation, male sex; province; facility size, type, and setting; and disclosure of HIV status at treatment initiation were associated with mortality (Table 2).

**Table 2:** Predictors of mortality among all antiretroviral therapy (ART) users and new ART users.

Predictors		Patients starting ART during the 2-year observation period (new ART users)				Patients already on ART at the start of the 2-year observation period (all ART users)			
		Model with time-zero patient characteristics		Model including time-varying retention in care		Model with time-zero patient characteristics		Model including time-varying retention in care	
		HR (95% CI)	*p*-value	HR (95% CI)	*p*-value	HR (95% CI)	*p*-value	HR (95% CI)	*p*-value
**Gender**	**Female**	1	0.027	1	0.057	1	0.012	1	0.052
	**Male**	1.86 (1.07–3.21)		1.70 (0.98–2.95)		1.82 (1.14–2.90)		1.59 (1.00–2.55)	
**Age (per year)**		1.01 (0.98–1.03)	0.537	1.01 (0.99–2.95)	0.274	1.00 (0.98–1.03)	0.780	1.01 (0.99–1.04)	0.356
**Initiation CD4 count (cells/μL)**	**≥200**	1	<0.001	1	<0.001	1	0.279	1	0.305
	**<200**	3.82 (2.08–7.03)		3.64 (1.92–6.91)		1.30 (0.81–2.11)		1.30 (0.79–2.13)	
**WHO stage**	**1**	1	0.006	1	0.025	1	0.611	1	0.361
	**2**	2.04 (1.04–3.99)		1.82 (0.87–3.78)		1.04 (0.54–2.00)		1.10 (0.56–2.17)	
	**3**	1.59 (0.78–3.25)		1.56 (0.75–3.23)		0.96 (0.54–1.71)		0.92 (0.51–1.66)	
	**4**	5.08 (1.97–13.09)		4.74 (1.71–13.14)		0.47 (0.15–1.48)		0.42 (0.15–1.19)	
**Province**	**Lusaka**	1	0.302	1	0.322	1	0.012	1	0.016
	**Eastern**	1.85 (0.92–3.75)		1.92 (0.91–4.05)		1.83 (1.07–3.13)		1.18 (1.04–3.16)	
	**Southern**	1.03 (0.55–1.92)		1.03 (0.53–2.02)		1.78 (1.04–3.05)		1.92 (1.12–3.30)	
	**Western**	1.88 (0.74–4.76)		1.84 (0.72–4.70)		2.73 (1.32–5.65)		2.57 (1.20–5.49)	
**Facility size (per 1,000 patients)**		0.99 (0.99–0.99)	0.012	0.99 (0.99–0.99)	0.020	0.99 (0.99–0.99)	0.039	0.99 (0.99–1)	0.087
**Facility setting**	**Urban**	1	0.181	1	0.361	1	0.049	1	0.176
	**Periurban**	1.11 (0.49–2.48)		1.10 (0.46–2.63)		0.64 (0.28–1.46)		0.78 (0.33–1.88)	
	**Rural**	0.47 (0.19–1.16)		0.57 (0.23–1.42)		0.44 (0.22–0.86)		0.51 (0.25–1.04)	
**Facility type**	**Health center**	1	0.046	1	0.087	1	0.025	1	0.069
	**Hospital**	0.61(0.38–0.99)		0.63 (0.37–1.07)		0.61 (0.39–0.94)		0.67 (0.43–1.03)	
**Disclosure of HIV status at enrolment**	**No**	1	0.424	1	0.637	1	0.007		0.013
	**Yes**	0.61 (0.19–2.03)		0.72 (0.18–2.83)		0.24 (0.08–0.67)		0.25(0.08–0.75)	
**History**	**Engaged, never late, never lost**	~	~	1	<0.001	~	~		<0.001
	**Engaged, previously late or lost**	~	~	0.17 (0.04–0.74)		~	~	0.92 (0.49–1.74)	
	**Late, never late or lost**	~	~	2.57 (1.04–6.36)		~	~	0.35 (0.79–1.56)	
	**Late, previously late or lost**	~	~	5.44 (1.17–25.29)		~	~	3.65 (1.53–8.68)	
	**Lost**	~	~	4.17 (1.59–10.93)		~	~	7.18 (3.73–13.78)	

**Fig 6:**
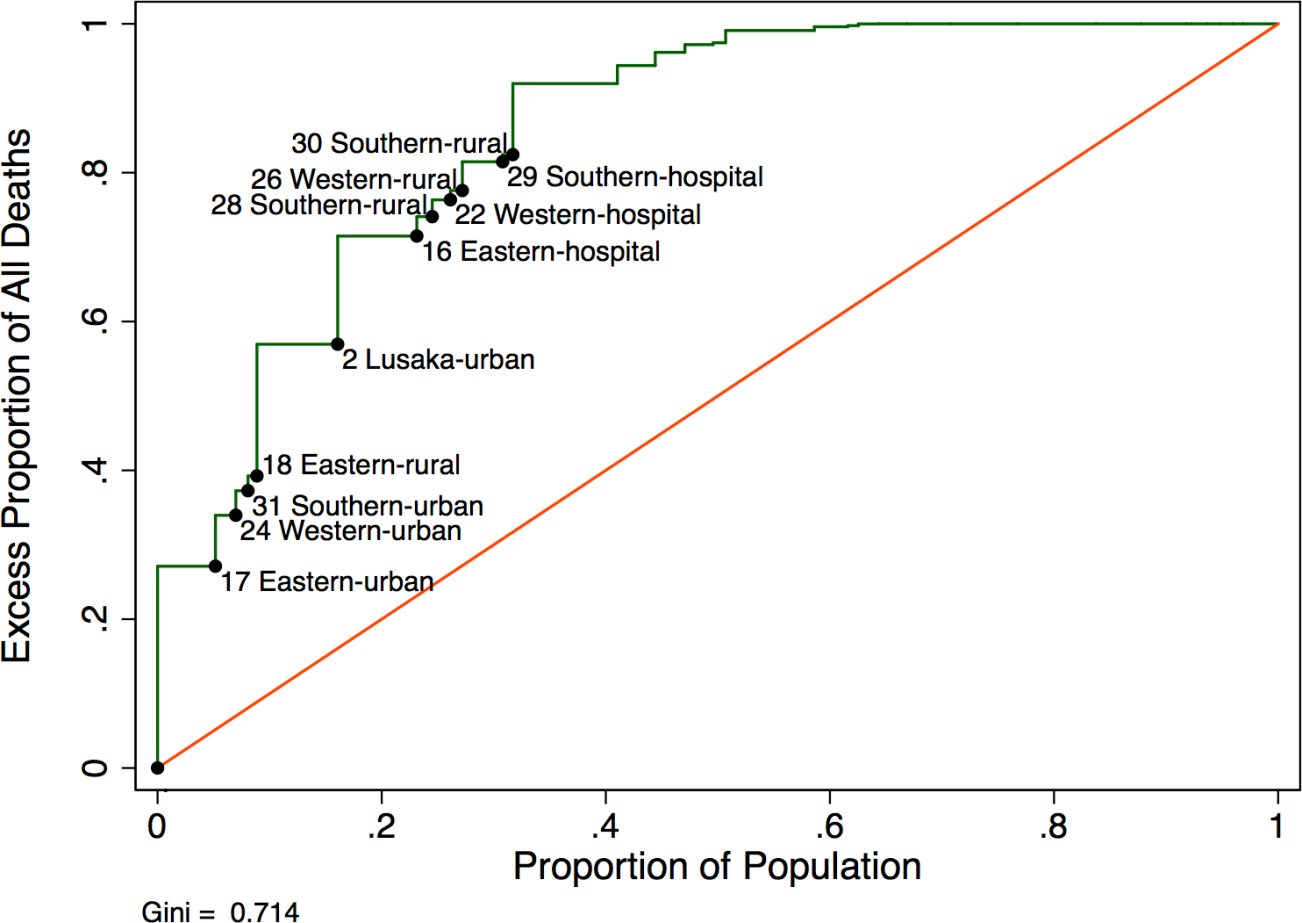
Modified Lorenz curve depicting excess mortality in new antiretroviral therapy (ART) users (*N* = 49,129).

A modified Lorenz curve to capture how “concentrated” mortality was across facilities showed that approximately half (57%) of excess deaths occurred in 15% of the population (Fig 6). The Gini coefficient was 0.714, suggesting that 71.4% of deaths were not evenly distributed across the population.

Mortality in all ART users was highest during the first year of treatment at 4.6 per 100 person-years (95% CI 4.0–5.5), fell to 2.9 per 100 person-years (95% CI 2.1–3.9) in the second year, and then remained steady at approximately 1.6% per year for up to 8 years on treatment. The overall mortality rate in this group was 1.7 per 100 person-years (95% CI 1.5–2.0), and a test of difference in mortality rates by year on treatment for up to 10 years was not statistically significant (*p*-value = 0.46). When examined alone, patients who started ART during the observation time for this study (new ART users) displayed the highest mortality in the first 90 days of treatment, at 7.9 per 100 person-years (95% CI 6.3–10.0); this fell to 4.6 per 100 person-years from 91–180 days and then fell to 3.3 per 100 person-years (95% CI 2.0–5.8). Regression models with time-varying factors capturing changing retention status found that being late and being lost were both associated with mortality, but a history of lateness or loss among those in care did not predict mortality after adjustment for other sociodemographic and clinical factors. These associations were more pronounced among all ART users than among new ART users (Table 2).

### Proportion of current deaths in windows of time on treatment

We estimated that 38.1% (95% CI 33.1%–43.4%) of mortality occurred among individuals in their first year on treatment, while the remaining 61.8% (95% CI 56.6%–66. 9%) was distributed among patients who had been on treatment for longer periods of time (Table 3). For patients newly starting treatment, 50.3% (95% CI 42.4%–58.2%) of all deaths occurred among those who had no history of missed visits, while 14.0% (95% CI 9.2%–20.7%) occurred in individuals while they were late, and 31.7% (95% CI 24.5%–39.9%) occurred among individuals while they were lost (S2 Fig). For those already on ART in the current period, 8.8% (95% CI 6.4%–12.1%) of deaths occurred among those who were always in care at the time of death, 13.7% (95% CI 9.7%–18.9%) occurred in those with a history of lateness (but who were in care at the time of death), 29.1% (95% CI 23.1%–35.9%) occurred in those who were in care but had a history of being lost, 11.7% (95% CI 8.1%–16.7%) occurred in individuals while they were late, and another 36.7% (95% CI 30.1%–43.8%) occurred among individuals while they were lost (S2 Fig).

**Table 3:** Duration on antiretroviral therapy (ART) and contribution to overall mortality among all ART users.

Time since ART initiation	Total person-years	Mortality rate			Proportion of total deaths		
		Estimate	95% CI		Estimate	95% CI	
			Lower	Upper		Lower	Upper
<1 year	43,015	4.6	4.0	5.5	0.4	0.3	0.4
1 to <2 years	32,572	2.9	2.1	3.9	0.2	0.1	0.2
2 to <3 years	27,049	1.6	1.1	2.3	0.1	0.1	0.1
3 to <6 years	66,261	1.6	1.3	2.1	0.2	0.2	0.3
6 to <8 years	30,439	1.6	1.1	2.2	0.1	0.1	0.1
8+ years	18,365	1.9	1.2	3.1	0.1	0.0	0.1

## Discussion

Despite the success of implementing HIV treatment in high-burden settings in Africa, we have identified high rates of mortality among those on treatment in Zambia, substantial under-reporting of deaths, and marked heterogeneity among provinces and sites. We applied for the first time, to our knowledge, a multistage sampling approach that included a sample of both sites and patients lost to follow-up in order to develop both provincially representative and site-level revised mortality estimates for an HIV program. In a sample representing over 160,000 patients across 4 provinces in Zambia, we found that 7% of all patients and 8% of new ART starters died within 2 years—from 10- to 20-fold higher than treated patients in Europe [18]. Provincial mortality rates developed from routine program data under-estimated true mortality rates in the provinces for new ART users by 3- to 9-fold, and revised mortality rates revealed the highest mortality rates for the HIV program in Western Province, followed by Southern, Eastern, and Lusaka. We identified important patient characteristics associated with short-term (e.g., CD4 level at initiation and male sex) and long-term mortality (e.g., male sex and disclosure), as others have described [9,19]. At the site level, care in a hospital and care in a nonurban setting were associated with a protective effect on mortality risk for all ART users, and the size of the facility had a statistically significant but modest protective effect. Importantly, we also found that the facility itself (regardless of province) is a critical driver of survival: even after adjustment for patients and facility characteristics, mortality across facilities ranged from 0 per 100 person years to 13.4 per 100 person years, even though the facilities in theory receive similar support (e.g., human resources and infrastructure). We also found that although mortality rates were highest in the first months of treatment, death rates among those on treatment for longer periods of time remain steady and high. None of these epidemiological observations would be possible using routinely collected death data, which differed markedly from sample-revised estimates.

Although mortality from childbirth, road accidents, and other factors are indeed higher in Africa, over 95% of the deaths ascertained in this cohort were due to “illness” rather than death from these competing causes. We believe the potential drivers of deaths include unmet needs for more advanced medical care, especially given the protective effect in our multivariable model of being treated in a hospital versus a health center, which may indicate a better capacity for advanced diagnostics and care in hospital-based clinics. Indeed, approximately half of deaths occur relatively shortly after a clinic visit. In a previous analysis, we found that the majority of patients who died after a recent visit had some opportunity for medical intervention that was missed—a more detailed assessment of the clinical encounters before death in these patients should be urgently undertaken [13]. At a time when public health approaches are focused on deintensifying care through differentiated service delivery [20,21], it should be recalled that in many cases, improving outcomes means identifying appropriate patients in whom to escalate care.

The provincially representative nature of the study enabled a new and more nuanced understanding of regional mortality among those newly starting ART and among the cross-section of all ART users in a province. First, the ratio of the revised mortality rate versus the naïve rate was highest in Lusaka Province—8-fold higher for all ART users and 9-fold higher for new ART users—compared with the other 3 provinces (ratios between 3–5). One interpretation of this is that populations in dense urban population centers such as Lusaka may be more likely to have their mortality under-reported. Lusaka Province is dominated by the capital city and transport hub of Lusaka, which has an urban population of 2.4 million people. It is conceivable that high mobility and/or fewer community linkages to health facilities result in less passive reporting of mortality than in the less urban provinces. The highest absolute revised mortality rates were in Western Province, which is a largely rural floodplain of the Zambezi River with the lowest population density of the 4 provinces, an HIV prevalence equal to that in Lusaka Province (16%), and limited economic activity other than cattle farming. Although our overall multivariable analysis suggested an association of nonurban facility setting with reduced mortality, health services in much of Western Province region are notoriously difficult to deliver and up-referrals for advanced care can be particularly challenging in the rainy season because of flooding and long distances to higher-level facilities. We believe these and potentially other factors may account for the high revised mortality rates in Western Province, although further investigation is needed at the provincial and site level. Indeed, the differences between the revised provincial mortality rates and between the ratios of the naïve and corrected morality rates in the provinces serve as starting points for both quality improvement work by provincial health offices and for further study. For example, future investigation could examine the system- and human-level causes of low levels of passive mortality reporting that we found across all 4 provinces, but particularly in Lusaka Province, and could investigate differential utilization of up-referral for sick patients by province and facility type and the association of provincial mortality rates with the quality and availability of advanced care services in the region.

Our results, drawn from a sample of patients visiting HIV treatment clinics between 2013 and 2015, indicate that even in an era of expanded eligibility for therapy and rising CD4 cell count thresholds, CD4 cell counts remain low at initiation and are a risk factor for mortality [22,23]. With clinical guidelines in Zambia now recommending universal treatment, we expect CD4 cell counts at treatment initiation will increase and mortality rates will decline overall. However, we anticipate that inconsistent healthcare utilization will continue to be a threat that could undermine potential gains. Although mortality amongst samples of lost patients has declined in recent years [11], perhaps reflecting an overall increase in CD4 cell counts at treatment initiation, our study also demonstrated that death rates remain unacceptably high even after 1 or more years of therapy, a time period when many might assume patients to be stable. An elevated risk of death, even years after initiating ART, was also seen in South African cohorts with high ascertainment of mortality through their national vital status registry. Standardized mortality ratios (SMRs) (observed deaths divided by the number of deaths expected if all patients were HIV negative) demonstrated that men in particular had elevated SMRs even after 48 months of therapy and having started therapy with a CD4 cell count >200 cells/mm^3^; women’s rates approached those of HIV-uninfected individuals by 48 months [4], a gender differential that has been widely noted [19]. These observations problematize a number of assumptions. First, it has been assumed that most patients on treatment for longer periods of time will be stable and can be placed in deintensified, demedicalized groups that emphasize social support and community-based treatment. Yet, high mortality rates in this group suggest that time on treatment itself cannot be a reliable marker of stability. Furthermore, it is assumed that intensive adherence support is critical only at initiation for most patients. Persistent deaths after 2 years on treatment imply that a subset of patients is poorly adherent or retained and that progression of HIV disease on treatment appears to be common. Therefore, effective support for adherence that is attuned to mitigating threats to adherence over time remains a crucial issue [24]. Previous work has suggested that adherence, as measured by medical possession ratio, is not optimal [25], and the high death rates on treatment may reflect disease progression. Finally, treatment does not fully protect individuals against tuberculosis; thus, rolling out isoniazid preventative therapy is a priority [26].

The marked heterogeneity of mortality across facilities is a crucial and novel observation made possible by our sampling scheme. The fact that mortality differs across facilities, even when higher-level systems support (e.g., supply chain, information systems, and central laboratory services) is relatively uniform, implies that the next generation of improvement efforts should be focused at the facility level and with access to improved data for better decision-making. In South Africa, where facilities are now linked to the national vital status registry, this is already possible, but in most settings in the region, it is not. Another implication of the site-to-site variability is that attention should be targeted to the high-volume, high-mortality-rate facilities because the absolute reductions in mortality are likely to be the biggest through improvements at these sites. Third, the presence of high-performing facilities suggests that strategies such as exemplars by positive deviants—in which high-performing facilities are brought in to assess and guide lower-performing facilities—should be explored [27]. Unexplained variability in small geographical areas supported by largely uniform systems spurred a generation of improvement science in the United States through the Dartmouth Atlas and other investigators and should be a crucial driver of improvement in the AIDS response as well [28,29].

Overall, our findings also imply that in Zambia and other similar settings, even if the regular use of site and subnational region-level mortality data for improvement were attempted, inaccuracy in the underlying data would undermine the utility of the approach. As we have shown, under-recording of mortality is the norm, and variability is masked under typical approaches to monitoring and evaluation, as evidenced by the differences in the ordering of mortality burden by province with use of naïve versus revised mortality rates. Although Demographic and Health Surveys (DHSs) and other door-to-door surveys provide representative data that can be used to evaluate trends in mortality, they are often not linked to sites and are not conducted with the frequency required to monitor for program performance and to measure the proximal effects of health systems interventions [30]. We propose 2 potential approaches to this issue. The first is to renew existing calls for nationwide death and birth registries, an approach successfully taken to scale in South Africa [31] and now being piloted in Zambia. Where this is not immediately possible, a scalable version of the representative multistage sampling performed in this study would be a feasible approach to incorporate into regional surveillance. Indeed, this approach is currently being adapted for broader use in Zambia, and we will shortly be publishing a cost analysis and a toolkit to encourage its broader use in the region. If efforts to expand this approach to improving our understanding of mortality are successful and supported by governments and funders, we would ideally be able to equip every site, district, and country with up-to-date access to accurate mortality data. In addition, incorporation of corrected provincial mortality rates into projection models could further improve their ability to inform resource needs and epidemic trends.

### Limitations

The methodology is predicated on tracing a random sample of individuals lost to care. We were unable to ascertain the vital status of about one-quarter of those lost and traced, which could have biased our results. However, we did not find differences in the sociodemographic or clinical characteristics among those who were traced and found compared with those we were unable to find. We were also not able to adjudicate causes of death and therefore are unable to distinguish clearly all-cause mortality versus HIV-related mortality. Further studies are needed to better understand clinical causes of death among HIV-infected individuals on ART in Zambia. Lastly, although the study included a comprehensive sample of Ministry of Health (MOH) ART sites in the 4 provinces, faith-based and other sites were not included in the estimates.

### Conclusions

To our knowledge, this study is the first to apply a multistage sampling-based approach to generating provincially representative mortality rates for HIV programs in low- and middle-income countries (LMICs). In addition to demonstrating that provincial mortality is substantially under-reported and variable among HIV-infected individuals on ART, we found unexpectedly high site-to-site variability in mortality after starting HIV treatment under routine health service delivery conditions. This heterogeneity among sites suggests that service delivery has much room to improve and that greater attention to systematically generating and using corrected mortality rates is a logical next step for ongoing quality improvement for national programs and major donors. In addition, given the associations of late/missed visits with mortality, greater attention to patient-centered and differentiated care approaches that are also sensitive to the need for care intensification may have a role in not only improving retention but addressing the markedly high death rates, despite the widespread use and availability of ART.

## Supporting information

S1 Fig Sampling flow diagram. (TIF)

S2 Fig Timing of deaths by care status and prior healthcare utilization patterns among (A) new antiretroviral therapy (ART) initiators and (B) all ART users.
(TIF)

S1 Table Tracing outcomes by clinic. (DOCX)

S2 Table Cumulative proportion of engagement states by duration of time on antiretroviral therapy (ART). (DOCX)

S3 Table Informant reported causes of death. (DOCX)

S4 Table Naïve and revised provincial mortality rate estimates among (A) new antiretroviral (ART) initiators and (B) all ART users.
(DOCX)

## Abbreviations

ART: antiretroviral therapy
CIDRZ: Centre for Infectious Disease Research in Zambia
DHS: Demographic and Health Survey
LMICs: low- and middle-income countries
MOH: Ministry of Health
SMR: standardized mortality ratio
UNAIDS: Joint United Nations Programme on HIV/AIDS
