# Supplementary figures and images for: Estimated mortality on HIV treatment among active patients and patients lost to follow-up in 4 provinces of Zambia: Findings from a multistage sampling-based survey
Source: PLoS Med. 2018 Jan 12;15(1):e1002489. doi: 10.1371/journal.pmed.1002489 (PMC5766235; doi:10.1371/journal.pmed.1002489)

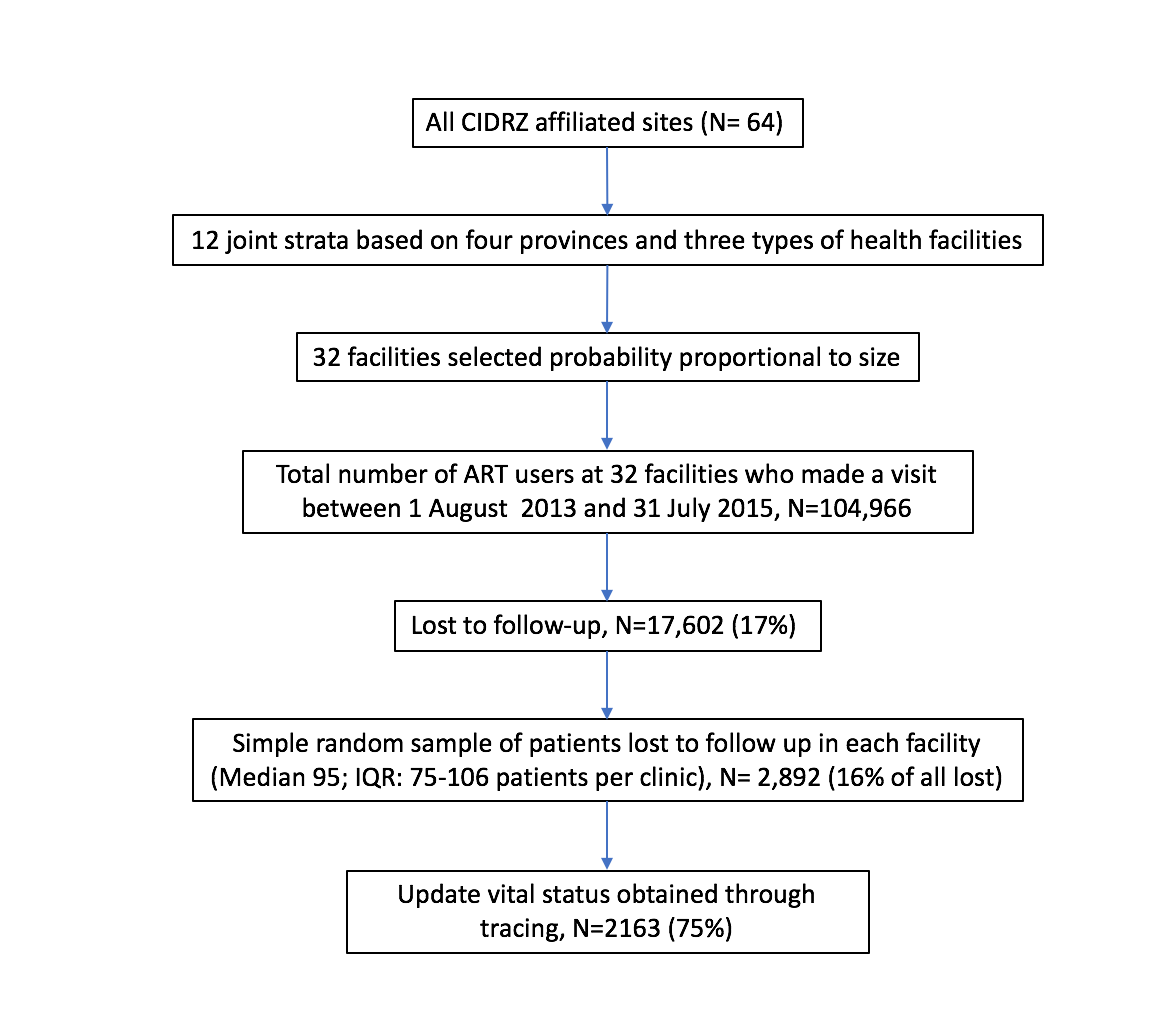

Supplement: S1 Fig — (TIF) [file pmed.1002489.s001.tif]

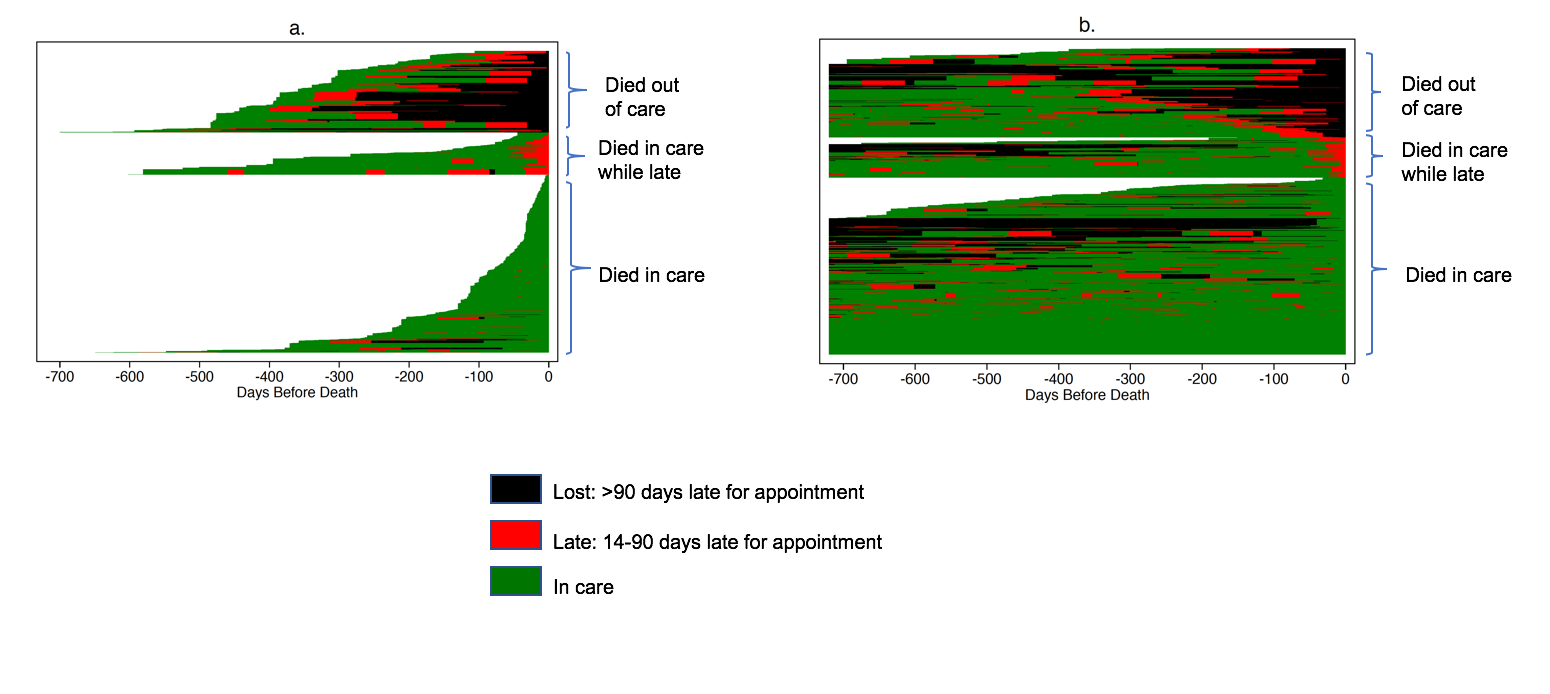

Supplement: S2 Fig — Timing of deaths by care status and prior healthcare utilization patterns among (A) new antiretroviral therapy (ART) initiators and (B) all ART users. (TIF) [file pmed.1002489.s002.tif]
