# Supplementary material for: Estimated mortality on HIV treatment among active patients and patients lost to follow-up in 4 provinces of Zambia: Findings from a multistage sampling-based survey
Source: PLoS Med. 2018 Jan 12;15(1):e1002489. doi: 10.1371/journal.pmed.1002489 (PMC5766235; doi:10.1371/journal.pmed.1002489)
Supplement: S1 Table — (DOCX) [file pmed.1002489.s003.docx]

| **Site** | **Total patients** | **Lost** | **Traced** | **Successful ascertainment of**  **vital status** |
| --- | --- | --- | --- | --- |
|  |  | N (%) | N (%) | N (%) |
| **1 Lusaka-urban** | 3284 | 759 (23) | 89 (12) | 50 (56) |
| **2 Lusaka-urban** | 8402 | 1205 (14) | 149 (12) | 115 (77) |
| **3 Lusaka-rural** | 3485 | 909 (26) | 150 (17) | 103 (69) |
| **4 Lusaka-urban** | 8241 | 2252 (27) | 150 (7) | 107 (71) |
| **5 Lusaka-urban** | 3757 | 1106 (29) | 150 (14) | 100 (67) |
| **6 Lusaka-hospital** | 1306 | 206 (16) | 119 (58) | 92 (77) |
| **7 Lusaka-hospital** | 1319 | 216 (16) | 118 (55) | 80 (68) |
| **8 Lusaka-urban** | 7307 | 2043 (28) | 117 (6) | 63 (54) |
| **9 Lusaka-urban** | 4720 | 2009 (43) | 144 (7) | 100 (69) |
| **10 Lusaka-urban** | 10599 | 2050 (19) | 140 (7) | 96 (69) |
| **11 Lusaka-rural** | 1564 | 797 (51) | 148 (19) | 127 (86) |
| **12 Lusaka-urban** | 4054 | 1119 (28) | 148 (13) | 117 (79) |
| **13 Lusaka-urban** | 3067 | 759 (25) | 147 (19) | 99 (97) |
| **14 Lusaka-urban** | 4734 | 929 (20) | 148 (16) | 80 (54) |
| **15 Eastern-urban** | 1031 | 335 (32) | 147 (44) | 138 (94) |
| **16 Eastern-hospital** | 7961 | 978 (12) | 150 (15) | 116 (77) |
| **17 Eastern-urban** | 6183 | 938 (15) | 143 (15) | 110 (77) |
| **18 Eastern-rural** | 957 | 72 (8) | 72 (100) | 59 (82) |
| **19 Eastern-hospital** | 3355 | 522 (16) | 148 (28) | 115 (78) |
| **20 Eastern-rural** | 1694 | 261 (15) | 150 (57) | 129 (86) |
| **21 Western-rural** | 497 | 155 (31) | 148 (95) | 137 (93) |
| **22 Western-hospital** | 1791 | 286 (16) | 150 (52) | 122 (81) |
| **23 Western-hospital** | 8922 | 2267 (25) | 149 (7) | 102 (68) |
| **24 Western-urban** | 2044 | 200 (10) | 122 (61) | 94 (77) |
| **25 Western-urban** | 1117 | 265 (24) | 125 (47) | 77 (62) |
| **26 Western-rural** | 1289 | 212 (16) | 125 (59) | 90 (72) |
| **27 Southern-hospital** | 4325 | 772 (18) | 149 (19) | 103 (69) |
| **28 Southern-rural** | 1522 | 157 (10) | 148 (94) | 137 (93) |
| **29 Southern-hospital** | 4096 | 511 (12) | 150 (29) | 101 (67) |
| **30 Southern-rural** | 1014 | 69 (7) | 69 (100) | 66 (96) |
| **31 Southern-urban** | 1337 | 312 (23) | 150 (48) | 127 (85) |
| **32 Southern-urban** | 2171 | 607 (28) | 150 (25) | 105 (70) |

S1 Table: Tracing outcomes by clinic
