# Supplementary material for: Estimated mortality on HIV treatment among active patients and patients lost to follow-up in 4 provinces of Zambia: Findings from a multistage sampling-based survey
Source: PLoS Med. 2018 Jan 12;15(1):e1002489. doi: 10.1371/journal.pmed.1002489 (PMC5766235; doi:10.1371/journal.pmed.1002489)
Supplement: S2 Table — (DOCX) [file pmed.1002489.s004.docx]

| **Engagement status** | **Days on ART** | **Cumulative Incidence of mortality** | **Lower CI** | **Upper CI** |
| --- | --- | --- | --- | --- |
| Died in care, never late & never lost | 90 | 0.015 | 0.014 | 0.016 |
| Died in care, previously late | 90 | 0 | 0 | 0 |
| Died in care, previously lost | 90 | 0 | 0 | 0 |
| Died out of care, never late & never lost | 90 | 0.006 | 0.006 | 0.007 |
| Died out of care, previously late | 90 | 0 | 0 | 0 |
| Died out of care, previously lost | 90 | 0 | 0 | 0 |
| Died in care, never late & never lost | 180 | 0.017 | 0.016 | 0.019 |
| Died in care, previously late | 180 | 0 | 0 | 0.001 |
| Died in care, previously lost | 180 | 0 | 0 | 0 |
| Died out of care, never late & never lost | 180 | 0.013 | 0.012 | 0.014 |
| Died out of care, previously late | 180 | 0 | 0 | 0 |
| Died out of care, previously lost | 180 | 0 | 0 | 0 |
| Died in care, never late & never lost | 365 | 0.02 | 0.019 | 0.021 |
| Died in care, previously late | 365 | 0.001 | 0.001 | 0.001 |
| Died in care, previously lost | 365 | 0.001 | 0 | 0.001 |
| Died out of care, never late & never lost | 365 | 0.023 | 0.022 | 0.024 |
| Died out of care, previously late | 365 | 0.002 | 0.002 | 0.003 |
| Died out of care, previously lost | 365 | 0 | 0 | 0.001 |
| Died in care, never late & never lost | 545 | 0.021 | 0.02 | 0.023 |
| Died in care, previously late | 545 | 0.002 | 0.002 | 0.003 |
| Died in care, previously lost | 545 | 0.001 | 0.001 | 0.001 |
| Died out of care, never late & never lost | 545 | 0.032 | 0.031 | 0.034 |
| Died out of care, previously late | 545 | 0.007 | 0.006 | 0.008 |
| Died out of care, previously lost | 545 | 0.001 | 0.001 | 0.002 |
| Died in care, never late & never lost | 730 | 0.022 | 0.02 | 0.023 |
| Died in care, previously late | 730 | 0.003 | 0.003 | 0.004 |
| Died in care, previously lost | 730 | 0.002 | 0.001 | 0.002 |
| Died out of care, never late & never lost | 730 | 0.036 | 0.034 | 0.037 |
| Died out of care, previously late | 730 | 0.009 | 0.008 | 0.01 |
| Died out of care, previously lost | 730 | 0.006 | 0.005 | 0.007 |
| Died in care, never late & never lost | 1095 | 0.023 | 0.021 | 0.024 |
| Died in care, previously late | 1095 | 0.004 | 0.004 | 0.005 |
| Died in care, previously lost | 1095 | 0.003 | 0.003 | 0.004 |
| Died out of care, never late & never lost | 1095 | 0.039 | 0.038 | 0.041 |
| Died out of care, previously late | 1095 | 0.013 | 0.012 | 0.014 |
| Died out of care, previously lost | 1095 | 0.013 | 0.012 | 0.015 |
| Died in care, never late & never lost | 1460 | 0.023 | 0.022 | 0.024 |
| Died in care, previously late | 1460 | 0.005 | 0.004 | 0.005 |
| Died in care, previously lost | 1460 | 0.005 | 0.005 | 0.006 |
| Died out of care, never late & never lost | 1460 | 0.041 | 0.039 | 0.043 |
| Died out of care, previously late | 1460 | 0.018 | 0.016 | 0.019 |
| Died out of care, previously lost | 1460 | 0.022 | 0.021 | 0.024 |
| Died in care, never late & never lost | 1825 | 0.023 | 0.022 | 0.025 |
| Died in care, previously late | 1825 | 0.005 | 0.004 | 0.005 |
| Died in care, previously lost | 1825 | 0.008 | 0.007 | 0.009 |
| Died out of care, never late & never lost | 1825 | 0.041 | 0.039 | 0.043 |
| Died out of care, previously late | 1825 | 0.02 | 0.018 | 0.021 |
| Died out of care, previously lost | 1825 | 0.031 | 0.029 | 0.033 |
| Died in care, never late & never lost | 2190 | 0.024 | 0.022 | 0.025 |
| Died in care, previously late | 2190 | 0.006 | 0.005 | 0.006 |
| Died in care, previously lost | 2190 | 0.01 | 0.008 | 0.011 |
| Died out of care, never late & never lost | 2190 | 0.042 | 0.04 | 0.044 |
| Died out of care, previously late | 2190 | 0.022 | 0.02 | 0.024 |
| Died out of care, previously lost | 2190 | 0.038 | 0.036 | 0.04 |
| Died in care, never late & never lost | 2555 | 0.024 | 0.022 | 0.025 |
| Died in care, previously late | 2555 | 0.006 | 0.005 | 0.007 |
| Died in care, previously lost | 2555 | 0.014 | 0.013 | 0.015 |
| Died out of care, never late & never lost | 2555 | 0.042 | 0.04 | 0.044 |
| Died out of care, previously late | 2555 | 0.024 | 0.022 | 0.025 |
| Died out of care, previously lost | 2555 | 0.044 | 0.042 | 0.046 |
| Died in care, never late & never lost | 2920 | 0.024 | 0.022 | 0.025 |
| Died in care, previously late | 2920 | 0.008 | 0.007 | 0.009 |
| Died in care, previously lost | 2920 | 0.015 | 0.014 | 0.017 |
| Died out of care, never late & never lost | 2920 | 0.043 | 0.041 | 0.045 |
| Died out of care, previously late | 2920 | 0.029 | 0.027 | 0.031 |
| Died out of care, previously lost | 2920 | 0.051 | 0.048 | 0.054 |
| Died in care, never late & never lost | 3285 | 0.024 | 0.022 | 0.025 |
| Died in care, previously late | 3285 | 0.008 | 0.007 | 0.009 |
| Died in care, previously lost | 3285 | 0.017 | 0.016 | 0.019 |
| Died out of care, never late & never lost | 3285 | 0.043 | 0.041 | 0.045 |
| Died out of care, previously late | 3285 | 0.03 | 0.028 | 0.032 |
| Died out of care, previously lost | 3285 | 0.06 | 0.057 | 0.063 |
| Died in care, never late & never lost | 3650 | 0.024 | 0.022 | 0.025 |
| Died in care, previously late | 3650 | 0.008 | 0.007 | 0.009 |
| Died in care, previously lost | 3650 | 0.021 | 0.019 | 0.023 |
| Died out of care, never late & never lost | 3650 | 0.044 | 0.042 | 0.046 |
| Died out of care, previously late | 3650 | 0.031 | 0.029 | 0.034 |
| Died out of care, previously lost | 3650 | 0.069 | 0.065 | 0.073 |

S2 Table: Cumulative proportion of engagement states by duration of time on ART
