# Supplementary material for: Estimated mortality on HIV treatment among active patients and patients lost to follow-up in 4 provinces of Zambia: Findings from a multistage sampling-based survey
Source: PLoS Med. 2018 Jan 12;15(1):e1002489. doi: 10.1371/journal.pmed.1002489 (PMC5766235; doi:10.1371/journal.pmed.1002489)
Supplement: S3 Table — (DOCX) [file pmed.1002489.s005.docx]

S3 Table. Informant reported causes of death

| Cause of death | N (%) |
| --- | --- |
| Accident | 32 (1.7%) |
| Illness | 961 (50.8%) |
| Related to childbirth | 2 (0.1%) |
| Suicide | 3 (0.2%) |
| Unknown | 893 (47.2%) |
| Total | 1891 |
