# Supplementary material for: Estimated mortality on HIV treatment among active patients and patients lost to follow-up in 4 provinces of Zambia: Findings from a multistage sampling-based survey
Source: PLoS Med. 2018 Jan 12;15(1):e1002489. doi: 10.1371/journal.pmed.1002489 (PMC5766235; doi:10.1371/journal.pmed.1002489)
Supplement: S4 Table — Naïve and revised provincial mortality rate estimates among (A) new antiretroviral (ART) initiators and (B) all ART users. (DOCX) [file pmed.1002489.s006.docx]

| Province | Naive mortality rate | | | Revised mortality rate | | |
| --- | --- | --- | --- | --- | --- | --- |
|  | Rate | Lower CI | Upper CI | Rate | Lower CI | Upper CI |
| Western | 1.77 | 1.49 | 2.1 | 8.73 | 6.4 | 11.93 |
| Southern | 2.11 | 1.79 | 2.49 | 7.42 | 6.04 | 9.12 |
| Lusaka | 0.53 | 0.45 | 0.62 | 4.56 | 3.56 | 5.84 |
| Eastern | 1.95 | 1.69 | 2.25 | 6.05 | 5.02 | 7.29 |

S4a Table: Naïve and revised provincial mortality rate estimates in new ART initiators

| Province | Naive mortality rate | | | Revised mortality rate | | |
| --- | --- | --- | --- | --- | --- | --- |
|  | Rate | Lower CI | Upper CI | Rate | Lower CI | Upper CI |
| Western | 0.81 | 0.73 | 0.9 | 3.32 | 2.97 | 3.7 |
| Southern | 0.83 | 0.75 | 0.92 | 2.66 | 2.45 | 2.89 |
| Lusaka | 0.24 | 0.22 | 0.27 | 2.02 | 1.82 | 2.25 |
| Eastern | 0.96 | 0.88 | 1.05 | 2.62 | 2.42 | 2.83 |

S4b Table: Naïve and revised provincial mortality rate estimates in all ART users
